# Diagnosis of an Infected Intrapulmonary Bronchogenic Cyst With an Emphasis on Lung Ultrasound

**DOI:** 10.7759/cureus.89631

**Published:** 2025-08-08

**Authors:** Soultana Foutzitzi, Panos Prassopoulos, Athanasios Chatzimichail, Savas P Deftereos

**Affiliations:** 1 Radiology, University Hospital of Alexandroupolis, Alexandroupolis, GRC; 2 Radiology, AHEPA University Hospital, Medical School, Aristotle University of Thessaloniki, Thessaloniki, GRC; 3 Pediatrics, Democritus University of Thrace, Alexandroupolis, GRC; 4 Radiology, Democritus University of Thrace/University General Hospital of Alexandroupolis, Alexandroupolis, GRC

**Keywords:** abscess, cxr, intrapulmonary bronchogenic cyst, lung ultrasound, pediatric

## Abstract

Lung ultrasound (LUS) is an increasingly valuable diagnostic modality for evaluating respiratory disorders in neonates and infants due to its rapid execution, ease of use, and, most importantly, absence of ionizing radiation. The sensitivity, cost-effectiveness, and clinical efficiency of LUS make it a key tool in supporting clinical decision-making and improving patient management. LUS demonstrates high diagnostic accuracy for identifying lung lesions in both infants and children, particularly lesions abutting the pleura. In this context, we highlight the diagnostic role of LUS in the accurate identification of a cystic pulmonary lesion during childhood. Postnatal LUS findings of an infected intrapulmonary bronchogenic cyst are presented and described in a 21-month-old female infant. Furthermore, these findings are compared across different radiologic modalities. Notably, LUS, computed tomography, and chest radiography show significant similarities in diagnosing an infected intrapulmonary bronchogenic cyst. LUS may serve as a reliable complementary or alternative imaging tool to chest radiography in the assessment of pediatric lung conditions and, in selected cases, could potentially replace unnecessary chest X-rays, thereby helping to reduce exposure to ionizing radiation in the pediatric population.

## Introduction

A bronchogenic cyst is an uncommon congenital anomaly that arises from abnormal budding of the embryonic foregut during early gestation. These lesions are typically benign and present as fluid-filled structures. While they are most often located in the mediastinum, a subset can occur within the lung parenchyma, known as intrapulmonary bronchogenic cysts. On rare occasions, they may be found in unusual locations such as the neck or subdiaphragmatic area [1,2].

Although these cysts may remain asymptomatic, particularly in infancy, they can cause clinical symptoms if complications develop [1]. The most common complication is secondary infection, which may present with nonspecific respiratory symptoms such as fever, cough, tachypnea, or respiratory distress. When infected, the cyst may contain air bubbles produced by microorganisms, creating an air-fluid level visible on imaging studies [2]. Other potential complications include compression of adjacent airways, hemorrhage, rupture, or, in very rare cases, malignant transformation [1-3]. Due to the fluid contents of such cysts, lung ultrasound (LUS) can be very useful to identify them. Furthermore, if complications exist, LUS is a sufficient method to reveal them in adjacent lung parenchyma. 

This report presents a case of an infected intrapulmonary bronchogenic cyst, with particular emphasis on the diagnostic value of lung ultrasound, which remains underutilized in such conditions.

## Case presentation

A 21-month-old female infant was referred to the hospital with signs of respiratory distress. The clinical course began five days before admission with fever, nasal congestion, and cough. On the third day of illness, worsening symptoms prompted a visit to a private pediatrician, who prescribed oral salbutamol syrup. Twenty-four hours prior to hospital presentation, the pediatrician modified the treatment to include inhaled salbutamol, oral cefprozil, and oral dexamethasone drops. Despite these interventions, the parents observed increasing tachypnea. Furthermore, an oxygen saturation (SpO₂) of 91% was documented by the pediatrician, prompting referral to the hospital for further evaluation and management.

The medical history indicated that the infant was full-term, delivered via cesarean section, and the first-born child from a monitored pregnancy, with a birth weight of 3150 g and length of 48 cm. Additionally, a bronchogenic cyst in the right upper lung (RUL), measuring 23 × 22 mm, had been identified on fetal ultrasonography during the third trimester. Prenatal ultrasound revealed a prominent fluid-filled cyst in the RUL. Postnatal chest X-ray showed a well-defined emphysematous area in the RUL, consistent with an “emphysematous-type cyst.” Based on these findings, a congenital pulmonary airway malformation was also considered.

**Figure 1:**
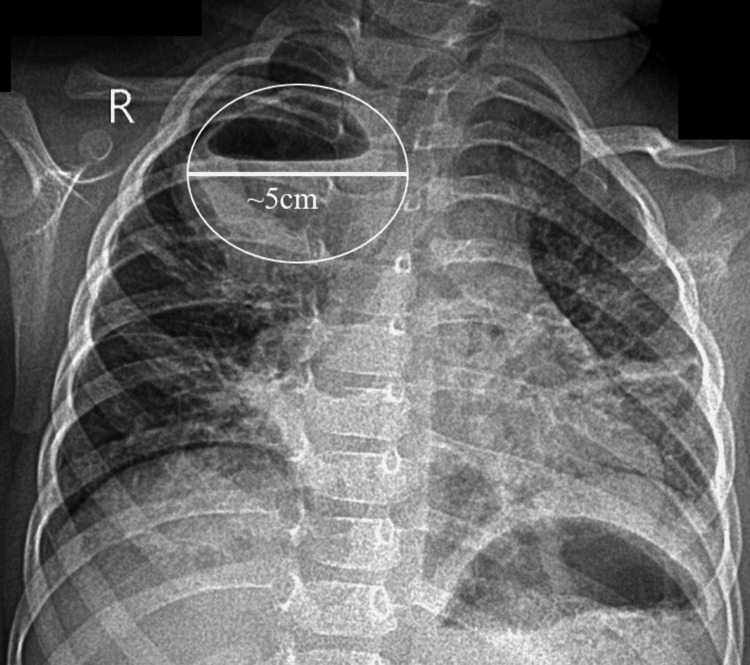
Initial Chest X-ray (CXR) Posteroanterior (PA) chest X-ray. There is a well-defined thick-walled ovoid lesion in the right upper lung (RUL) with an air-fluid level. The lesion’s max diameter is 5 cm. Also, peri-bronchial infiltrations and an atelectasis (left middle thoracic field) are present.

The initial chest X-ray (Figure 1) revealed a well-defined, thick-walled, ovoid lesion in the RUL containing an air-fluid level. The inferior margins of the lesion appeared unclear, presumably due to coexisting pulmonary consolidations in the right middle lung field. The diagnosis of lung abscess in the context of acute pneumonia was supported by clinical findings, including leukocytosis with neutrophilia, an elevated erythrocyte sedimentation rate, and a high C-reactive protein level (Table 1).

**Table 1:** Clinical and laboratory findings WBC/μl: White Blood Cell per microliter, NEUT: Neutrophils, ESR: erythrocyte sedimentation rate, CRP: C-Reaction Protein, SpO₂: oxygen saturation

Clinical Findings	Values	Reference Range
SpO₂	91% (by a pulse oximeter)	95% - 100%
Leucocytosis (neutrophilia)	WBC: 13280/μl (NEUT: 76.5%)	3500 - 10800/μl
ESR	13 mm/h	0-9 mm/h
CRP	5.27 mg/dl	<1.00 mg/dl

**Figure 2:**
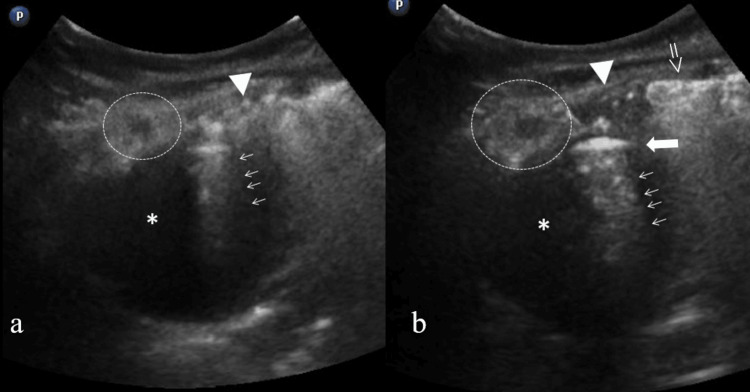
Lung ultrasound at the site of lesion Lung ultrasound (LUS) with prominent finding of an ovoid hypoechoic lesion (star: fluid-like appearance). Superiorly a dense hyperechoic arcuate-shaped area is present producing a comet-tail artifact (arrows) consistent with air having a linear inferior border (thick arrow in (b): air-fluid level). There is also a consolidated area (circle) with hypoechoic centre suspicious for necrotic pneumonia. Furthermore, superiorly to main lesion, in subpleural location, a hypoechoic area is present which looks like a jagged echoic edge and represents replaced by inflammatory exudate area, also known as “shred sign” (arrowhead). Finally, adjacent to “shred-sign” area is a hyperechoic subpleural region with extension throughout the image’s field, which represents “coalescent B-Lines” (open arrow in (b)).

The role of lung ultrasound is well established in interstitial lung disease as well as in peripheral consolidations. Furthermore, every intrapulmonary lesion could affect the pleura and the adjacent parenchyma at least [4,5]. Based on these, a subsequent LUS revealed a thick-walled, hypoechoic lesion with posterior acoustic enhancement, containing an air-fluid level in the RUL (Figure 2). The lesion was peripheral, abutting the pleura, and continuous with an area of consolidation. The consolidation itself exhibited central hypoechoic features, raising suspicion for coexisting necrotizing pneumonia. Color Doppler sonography showed no prominent vascularity. 

**Figure 3:**
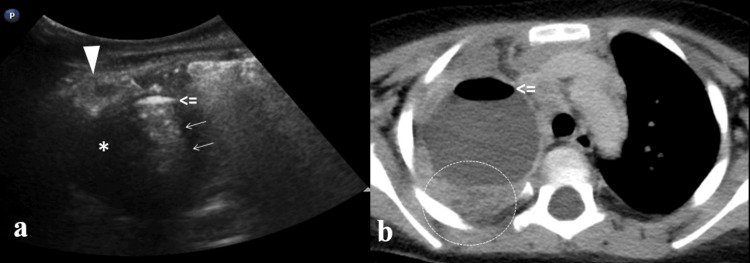
LUS and CT similarities Comparison between lung ultrasound (LUS) (a) and CT (b) examinations. There are a lot of similarities between the images according to depiction of air-fluid level (open arrow) and consolidation (circle). For a more extensive description please see Figure 2.

The computed tomography (CT) scan confirmed the LUS findings (Figure 3), revealing a large, oval-shaped lesion in the RUL with adjacent areas of consolidation. The lesion contained an air-fluid level, with a small amount of air located anteriorly, and showed a thick, enhancing wall. No communication with the tracheobronchial tree was identified in this examination. The coexisting consolidations in the RUL and in the apical segment of the right lower lobe exhibited small areas of low attenuation, consistent with necrotic pneumonia. A small associated ipsilateral pleural effusion was also noted. 

Based on the clinical and imaging findings, a diagnosis of an infected intrapulmonary bronchogenic cyst in the context of pneumonia was made, and a decision was taken to proceed with conservative antibiotic treatment.

**Figure 4:**
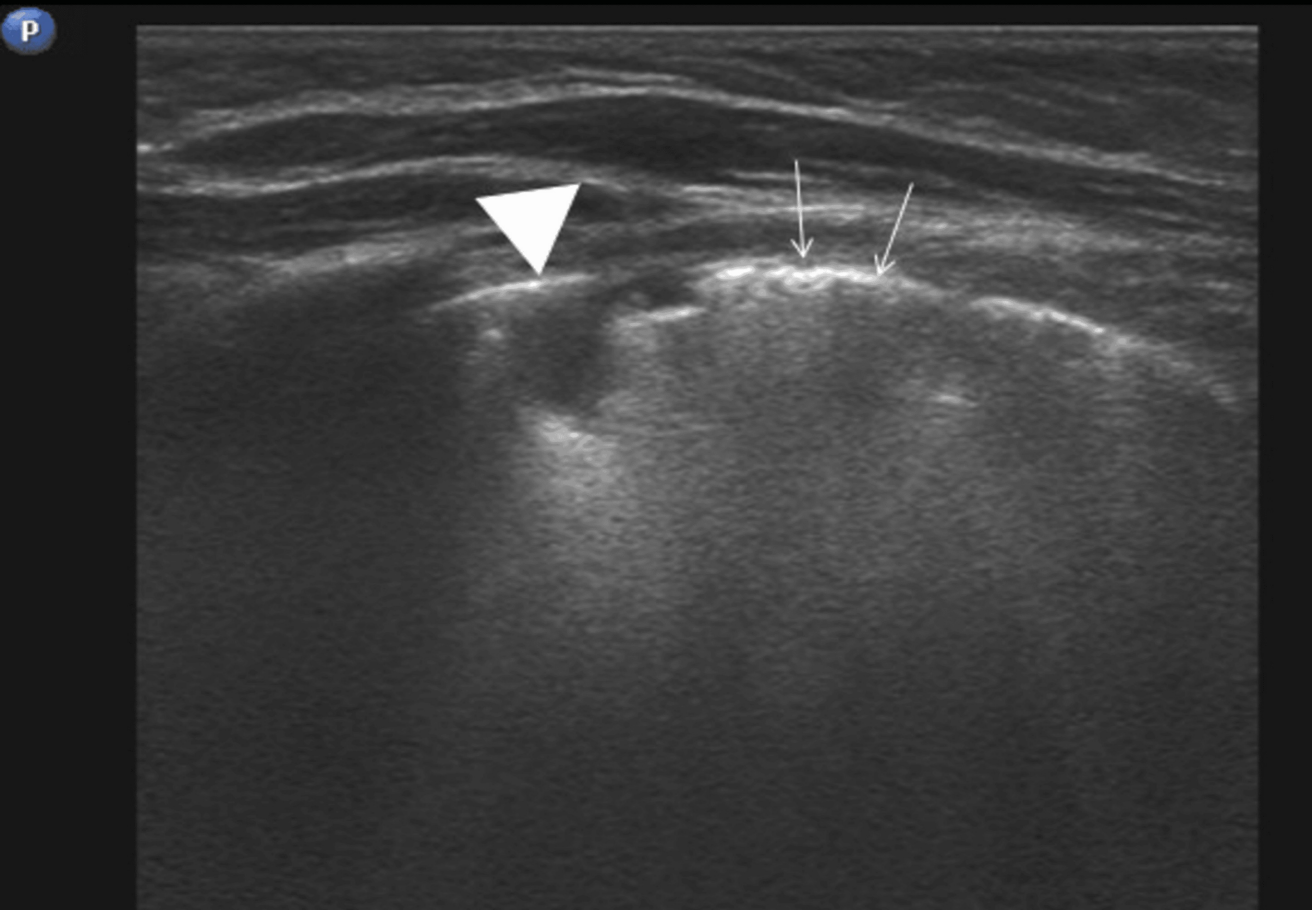
Follow-up LUS (one month) Follow-up lung ultrasound (LUS) reveals residual consolidation (arrowhead: “shred sign”) and coalescent B-Lines (arrows). The bronchogenic cyst is not visualised due its deeper location.

**Figure 5:**
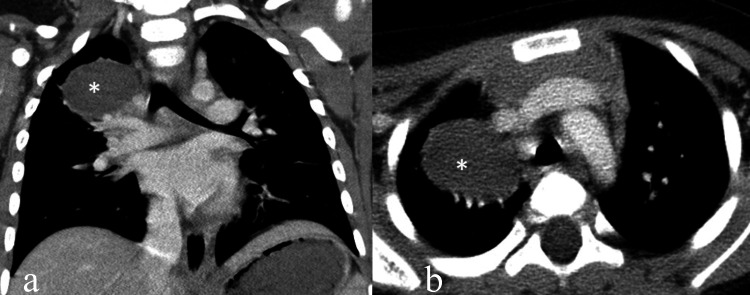
Follow-up CT (three months) Follow-up CT images in coronal (a) and axial (b) plane reveal cyst lesion (star) with no evidences of infection.

In the follow-up CT scan, the lesion showed a reduction in size. It now contained only fluid, and after intravenous contrast administration, mild enhancement of a thin wall was observed (Figures 4, 5).

## Discussion

Congenital pulmonary malformations can be either cystic or solid, encompassing a broad range of anomalies, including congenital cystic adenomatoid malformation, bronchopulmonary sequestration, bronchogenic cyst, congenital lobar emphysema, and segmental bronchial atresia [1,2].

A bronchogenic cyst is typically round and solitary, representing a congenital, non-vascular bronchopulmonary anomaly that is rarely encountered in pediatric practice [1]. It results from abnormal development of the respiratory tract during embryogenesis and may be located either intrapulmonary (within the lung parenchyma) or mediastinal. The cyst arises from an abnormality in bronchial embryogenesis, which occurs between the 20th and 40th day of intrauterine life, and it does not communicate with the tracheobronchial tree. Embryologically, it originates from an ectopic respiratory bud that fails to develop beyond its early embryonic stage [3]. Bronchogenic cysts can occasionally be multiple or multilobular; however, in our case, the cyst was solitary. Furthermore, bronchogenic cysts are asymptomatic in 5%-19% of cases [4,5], although most cases present with symptoms in infants and toddlers [6].

Chest X-ray, both at the initial presentation and at the end of treatment, was essential for establishing the diagnosis and monitoring the therapeutic response. LUS also contributed reliably to the diagnosis of the intrapulmonary bronchogenic cyst, both initially and during follow-up after treatment. When visualized by LUS, an intrapulmonary bronchogenic cyst typically appears as a round or oval hypoechoic lesion because it is filled with fluid. The cyst often presents with well-defined borders, which helps distinguish it from the surrounding tissue. A characteristic finding is posterior acoustic enhancement, commonly observed in fluid-filled lesions, resulting from the transmission of ultrasound waves through the cystic content - an imaging hallmark that enhances diagnostic confidence [7,8]. The absence of internal vascularity on color Doppler imaging further supports the diagnosis of a non-vascular, cystic anomaly and is highly suggestive of a fluid-filled structure, helping to differentiate it from solid or vascularized lesions. In cases where the cyst contains air, as seen in inflamed cysts, ultrasound artifacts such as comet-tail or ring-down artifacts may appear, complicating interpretation [8,9].

However, if the cyst is located deep within the lung parenchyma or is surrounded by normally aerated lung tissue, the utility of ultrasound becomes limited. The presence of overlying air significantly attenuates the ultrasound beam, preventing clear visualization of deeper lesions [10-12]. Nevertheless, a deeply located lesion often affects the pulmonary parenchyma, producing ultrasound findings - actually artifacts - known as B-lines. While these lines are not specific, they are highly sensitive and can be extremely useful for monitoring pulmonary pathology. 

LUS has become an increasingly valuable diagnostic tool in pediatric respiratory diseases because of its non-invasive nature, lack of radiation exposure, bedside applicability, and ability to provide dynamic imaging. LUS can still offer significant information when assessing cystic or fluid-filled pulmonary lesions, including intrapulmonary bronchogenic cysts [7,8].

The sonographic appearance of a bronchogenic cyst can resemble other pathological entities. The differential diagnosis includes lung abscesses, which typically present as a hypoechoic lesion, possibly with internal echoes if purulent material is present; cystic pneumonia, which may appear with mixed echogenicity and is often associated with consolidation; congenital cystic adenomatoid malformation, which may exhibit multiple cystic components of varying size and echotexture [13-15]; and necrotic pulmonary lesions, which can appear hypoechoic or heterogeneous due to liquefaction necrosis [4].

Accurate diagnosis requires correlation with clinical findings and complementary imaging modalities. Although CT remains the gold standard for detailed anatomical and pathological characterization [9,10,12,13], LUS offers a highly practical and repeatable tool for both initial assessment and follow-up, particularly in resource-limited settings or when serial imaging is necessary [7,8]. Its utility extends beyond diagnosis, serving as a valuable guide for interventional procedures such as aspiration or drainage when clinically indicated [7].

## Conclusions

In summary, LUS is a powerful adjunct in the diagnostic algorithm for bronchogenic cysts, combining safety, accessibility, and diagnostic accuracy. Its role in pediatric respiratory imaging continues to expand. Although CT remains the preferred imaging modality for diagnosing bronchogenic cysts, LUS may serve as an important alternative in pediatric patients and in selected cases. To the best of our knowledge, there is no case referred to in the literature. Thus, this present case highlights the valuable role of LUS as a non-invasive, bedside imaging modality in the diagnosis and follow-up of infected intrapulmonary bronchogenic cysts. LUS provided critical diagnostic information, such as the air-fluid level and associated consolidation, with excellent correlation to CT findings. 
